# Reversibility of cellular aging by reprogramming through an embryonic-like state: a new paradigm for human cell rejuvenation

**DOI:** 10.5195/cajgh.2013.88

**Published:** 2014-01-24

**Authors:** Jean-Marc Lemaitre

**Affiliations:** Director of INSERM Laboratory, Genome Plasticity and Aging, Institute of Functional Genomics, Montpellier, France

**Keywords:** embryonic stem cells, aging, longevity, cell rejuvination

## Abstract

Direct reprogramming of somatic cells into induced pluripotent stem cells (iPSCs) provides a unique opportunity to derive patient-specific stem cells with potential application in autologous tissue replacement therapies and without the ethical concerns of Embryonic Stem Cells (hESC). However, this strategy still suffers from several hurdles that need to be overcome before clinical applications. Among them, cellular senescence, which contributes to aging and restricted longevity, has been described as a barrier to the derivation of iPSCs. This suggests that aging might be an important limitation for therapeutic purposes for elderly individuals. Senescence is characterized by an irreversible cell cycle arrest in response to various forms of stress, including activation of oncogenes, shortened telomeres, DNA damage, oxidative stress, and mitochondrial dysfunction. To overcome this barrier, we developed an optimized 6-factor-based reprogramming protocol that is able to cause efficient reversing of cellular senescence and reprogramming into iPSCs. We demonstrated that iPSCs derived from senescent and centenarian fibroblasts have reset telomere size, gene expression profiles, oxidative stress, and mitochondrial metabolism, and are indistinguishable from hESC. Finally, we demonstrate that re-differentiation led to rejuvenated cells with a reset cellular physiology, defining a new paradigm for human cell rejuvenation. We discuss the molecular mechanisms involved in cell reprogramming of senescent cells.

